# MAVS Deficiency Is Associated With a Reduced T Cell Response Upon Secondary RSV Infection in Mice

**DOI:** 10.3389/fimmu.2020.572747

**Published:** 2020-10-06

**Authors:** Michelle Paulsen, Augusto Varese, Nawamin Pinpathomrat, Freja C. M. Kirsebom, Malte Paulsen, Cecilia Johansson

**Affiliations:** Respiratory Infections Section, St Mary’s Campus, National Heart and Lung Institute, Imperial College London, London, United Kingdom

**Keywords:** memory T cells, cytokines, respiratory viral infection, lung, innate immunity

## Abstract

Infections with respiratory syncytial virus (RSV) occurs repeatedly throughout life because sustained, protective memory responses fail to develop. Why this occurs is not known. During RSV infection the recognition of the virus *via* the cytosolic RIG-I like receptors and signaling *via* the adaptor protein MAVS is crucial for mounting an innate immune response. However, if this signaling pathway is important for T cell responses during primary infection and during re-infection is not fully elucidated. We describe a second peak of pro-inflammatory mediators during the primary immune response to RSV that coincides with the arrival of T cells into the lung. This second peak of cytokines/chemokines is regulated differently than the early peak and is largely independent of signaling *via* MAVS. This was concurrent with *Mavs*^−/−^ mice mounting a strong T cell response to primary RSV infection, with robust IFN-γ; and Granzyme B production. However, after RSV re-infection, *Mavs*^−/−^ mice showed fewer CD4^+^ and CD8^+^ short term memory T cells and their capacity to produce IFN-γ; and Granzyme B, was decreased. In sum, cytosolic recognition of RSV is important not only for initiating innate anti-viral responses but also for generating or maintaining efficient, short term T cell memory responses.

## Introduction

Respiratory syncytial virus (RSV) is the most common cause of lower respiratory tract infections (LRTI) in children, resulting in bronchiolitis and viral pneumonia and often leading to hospitalization (1, 2). Currently, no vaccines or effective treatments against RSV replication and induced disease are available with a pressing need to understand the underlying mechanisms for development of disease and how to obtain protection against the infection. Innate immune responses are important for an efficient, early anti-viral response (2, 3). Type I interferons (IFNs) are essential mediators of the innate immune response and their protective role during RSV infection has been demonstrated in animal models (4–7). Importantly, RSV disease severity has been linked to polymorphisms in genes that control the IFN system (8–10). Alveolar macrophages (AMs) are the primary source of type I IFNs during RSV infection and these type I IFNs lead to both cell intrinsic viral control and cell extrinsic anti-viral effects (5). One of the major cell extrinsic effect of type I IFNs is to trigger CCL2-induced recruitment of inflammatory monocytes, which contribute to the control of the virus (5). RSV infection of mice deficient in retinoic acid inducible gene I receptors (RLR) adaptor protein mitochondrial antiviral-signaling protein (MAVS; *Mavs*^−/−^) revealed a severe deficiency in type I IFNs, pro-inflammatory cytokines and chemokines and inflammatory monocyte recruitment during RSV infection with associated increased illness and viral burden (5, 6, 11). Early anti-viral cytokines are not only important to contain virus replication and spread, they also play an essential role for the induction and regulation of protective adaptive immune responses (12). T cell responses are important for the final clearance of RSV. However, the T cells also contribute to illness and disease severity during RSV infection (2, 13, 14). In the absence of most innate immune responses several reports suggest that *Mavs*^−/−^ mice are perfectly capable of inducing RSV-specific T cell responses (5, 6, 11). However, how viral sensing through the RLR-MAVS pathway influences short term T cell memory responses during RSV infection is not fully elucidated. Upon primary infection with a pathogen a population of long-lived memory T cells are maintained in the tissue and circulation in order to respond rapidly to potential secondary encounter with the same pathogen (15). In this study, we investigated the influence of innate immune responses on the initiation and effector function of T cells during the acute and memory phase of immune response to RSV infection. Following primary challenge with RSV, *Mavs*^−/−^ mice displayed cytokine/chemokine production as well as infiltration of immune cells including an increased T cell recruitment into the lung tissue and airways with heightened T cell effector functions compared to wild type (wt) mice. However, after re-infection, *Mavs*^−/−^ mice failed to mount a CD4^+^ or CD8^+^ T cell response to levels detected in wt mice. In addition, fewer of the memory T cells in the *Mavs*^−/−^ mice produced Granzyme B and IFN-γ. Our study demonstrates a dependency on the early innate immune responses for the presence of RSV-specific memory T cells during RSV re-infection.

## Materials and Methods

### Mice

Wt mice and mice deficient in MAVS (*Mavs*^−/−^), obtained from S. Akira [World Premier International Immunology Frontier Research Center, Osaka University, Osaka, Japan, (16)] were bred and maintained in specific pathogen-free conditions. Both strains were *Ifna6 ^gfp/+^* but since *Ifna6* expression was not a primary readout the mice are designated as wildtype (wt) and *Mavs*^−/−^ mice. The mice were gender- and age-matched (7–12 weeks) in each experiment. All animal experiments were reviewed and approved by Animal Welfare and Ethical Review Board (AWERB) within Imperial College London and approved by the UK Home Office in accordance with the Animals (Scientific Procedures) Act 1986 and the ARRIVE guidelines were followed.

### Virus and Infection

Plaque-purified human RSV (hRSV herein referred to as RSV) A2 (originally from ATCC) was grown in HEp-2 cells with DMEM supplemented with 2% fetal calf serum (FCS) and 2mM L-glutamine. Mice were lightly anesthetized and infected intranasally (i.n.) with 1.8x10^6^ FFU of RSV in 100 μl.

### Isolation of Cells From Airway (BAL) and Lung

Mice were sacrificed at different time points after infection. For some mice as indicated in figure legends, an intravenous injection of 2 μg anti-CD45-BUV394 (Biolegend, Cambridge, UK) in 200 μl was performed 10 min before euthanasia. Bronchoalveolar lavage (BAL) was performed by flushing the lungs 3 times with 1 ml phosphate-buffered saline (PBS) containing 0.5 mM EDTA (Life Technology, Paisley, UK). The obtained lavage was spun at 3,500 x *g* for 5 min to separate the BAL fluid from the cells. Lungs were perfused with PBS. To obtain single cell suspension, lungs were collected into C-Tubes (Milteny Biotech, Surrey, UK) containing complete DMEM (cDMEM; supplemented with 10% FCS, 2mM L-glutamine, 100 U/ml penicillin and 100 μg/ml streptomycin), 1 mg/ml Collagenase D (Roche, Welwyn Garden City, UK) and 30 μg/ml DNase I (Sigma Aldridge, Dorset UK) and processed with gentleMACS dissociator according to manufacturer’s protocol. After incubation for 30 min at 37°C the lungs were processed in the gentleMACS dissociator again. The red blood cells in BAL cells and lung single cell suspension were lysed using ACK buffer.

### Flow Cytometry

Lung or BAL cells were incubated for 20 min at 4°C with purified rat IgG2b anti-mouse CD16/CD32 receptor antibody and then stained with fluorochrome-conjugated antibodies against CD11c (HL3, PE-CF594), CD11b (M1/70, AF700), CD45 (30-F11, eFluor780 or BV605), Ly6C (HK1.4, eFluor450), CD103 (2E7, PerCP-Cy5.5), Siglec-H (eBio440c, eFluor660), B220 (RA3-6B2, eFluor450), Siglec-F (E50-2450, PE), CD64 (X54-5/7.1, APC), Ly6G (1A8, BV570), CD3 (17A2, AF700), CD4 (GK1.5, PE), CD8 (53-6.7, eFluor780), CD19 (6D5, FITC), CD44 (IM7, PE-Cy7), and CD62L (MEL-14, BV421) in PBS containing 1% BSA, 5 mM EDTA and 0.05% NaN_3_ for 25 min at 4°C. This was followed by an incubation with fixable live-dead Aqua dye (Invitrogen, Paisly, UK) for 30 min at 4°C before fixing the cells with fixation buffer (Biolegend, Cambridge, UK). Alexa Fluor 647-conjugated M_187-195_ tetramers (H-2D^b^/NAITNAKII) were obtained from the NIH Tetramer Core Facility (Emory University Atlanta, GA, USA). M Tetramer staining was performed following Fc-block and prior to surface staining for 30 min at RT in PBS containing 1% BSA, 5 mM EDTA, and 0.05% NaN_3_.

For intracellular staining the cells were re-stimulated with 5 μg/ml RSV M_187-195_-peptide for 1 h at 37°C. After addition of Golgi Plug (BD Biosciences) the samples were incubated for another 3 h, stained for surface marker expression as described above and fixed in fixation buffer (Biolegend). Cells were stained with fluorochrome-conjugated antibodies against granzyme B (GB11, PE-CF594) and IFN-γ (XMG1.2, BV711) in the presence of purified rat IgG2b anti-mouse CD16/CD32 receptor antibody in permeabilization buffer (Biolgend) for 60 min. Samples were measured on a Becton Dickinson Fortessa LSR-SORP equipped with 20 mW 355 nm, 50 mW 405 nm, 50 mW 488 nm, 50 mW 561 nm, 20 mW 633 nm lasers and a ND1.0 filter in front of the FSC photodiode. Acquisition was set to 250,000 single, live cells. PMT voltages were adjusted after standardized CST checks minimizing the spectral overlap to increase data precision. All antibodies were purchased from BD, eBioscience or Biolegend. Data were analyzed using FlowJo software (Treestar, Ashland, OR, USA).

### Chemokine, Cytokine, and RSV-Specific Ig Detection

BAL fluid was assessed using a granzyme B and IFN-γ ELISA kit (R&D Systems, Minneapolis, MN, USA) according to manufacturer’s instructions. Detection limits were 31 pg/ml and 16 pg/ml respectively.

To quantify RSV-specific IFN-γ levels from lung cells, 4x10^5^ lungs cells/well were stimulated with either medium, RSV (MOI 1) or 5, 0.5, and 0.05 ng/ml RSV M_187-195_ (H-2D^b^/NAITNAKII) peptide or as control, 5 ng/ml RSV M2_82-90_ (H-2K^d^/SYIGSINNI) in cDMEM for 72 h at 37°C. Supernatants were assessed using the IFN-γ ELISA kit (R&D Systems) according to manufacturer’s instructions.

To quantify chemokines and cytokines in the lung tissue, lungs were homogenized using a tissue lyser (Qiagen, Crawley, UK) in PBS containing protease inhibitor according to manufacturer’s instructions (Roche). After spinning for 10 min at 10,000 × *g* supernatants were analyzed using a Magnetic Cytokine 20-plex panel for Luminex (Life Technologies) according to manufacturer’s instructions. The data were obtained from a Bioplex 200 system (Bio-Rad laboratories).

To quantify RSV-specific Ig, in serum and BAL, ELISA was used. Briefly, ELISA plates were coated with RSV or HEp-2 control antigens in the same concentration and incubated overnight at 4°C. After blocking with 1% BSA in PBS for 1 h at 37°C, serum or BAL fluid was added in serial dilutions. After a further incubation for 2 h at 37°C HRP-conjugated detection antibodies were added; anti-total Ig (0.25µg/ml, Bio-Rad, Hertfordshire, UK) or anti- IgA antibodies (0.5µg/ml, Bio-Rad). The plates were incubated for 2 h at 37°C before adding TMB substrate (Life-Tech) and after achieving proper color development, the reaction was stopped using 2 M H_2_SO_4_. The plates were analyzed using a spectrophotometry (SoftMax Pro v5.2; Molecular Devices) and optical density was read at 450 nm. The data are plotted as absorbance (Abs) 450 of RSV-specific Ig minus HEp-2 specific Ig.

### RNA Isolation and Quantitative RT-PCR

Lungs were homogenized using a TissueLyser LT (Qiagen), and total RNA was extracted using RNeasy Mini kit including DNA removal as described by the manufacturer (Qiagen). 1 μg RNA was reverse transcribed using High Capacity RNA-to-cDNA kit (Applied Biosystems, Paisley, UK) according to manufacturer’s instructions. To quantify RNA levels in lung tissue, quantitative RT-PCR reaction for *Tnfa*, *Ifng*, and RSV L gene was performed using primers and probes as previously described (4) in QuantiTect Probe PCR Master Mix (Qiagen). For absolute quantification, the exact number of copies of the gene of interest was calculated using a plasmid standard, and the results were normalized to the housekeeping gene *Gapdh* (Applied Biosystems). The relative expression of *Il1b*, *Il6*, and *Cxcl1* (all from Applied Biosystems) to the housekeeping gene *Gapdh* was determined. First, the ΔCt (Ct=cycle threshold) between the target gene and *Gapdh* for each sample was calculated, following the calculation of 2^-ΔCt^. Analysis was performed using 7500 Fast System SDS Software (Applied Biosystems).

### Statistical Analysis

Statistical analysis of data was performed using GraphPad Prism 6 (GraphPad Software Inc., La Jolla, CA, USA). Two-group comparisons were performed using unpaired, two-tailed Student’s *t* test. One-way ANOVA with Tukey’s *post hoc* test was used to compare multiple groups. For all tests a value of P<0.05 was considered as significant. *p<0.05; **p<0.01; ***p<0.001; ND, not detectable.

## Results

### Inflammatory Cytokines and Recruitment of Innate Immune Cells During the Adaptive Response Is Independent of MAVS Signaling

**Figure 1:**
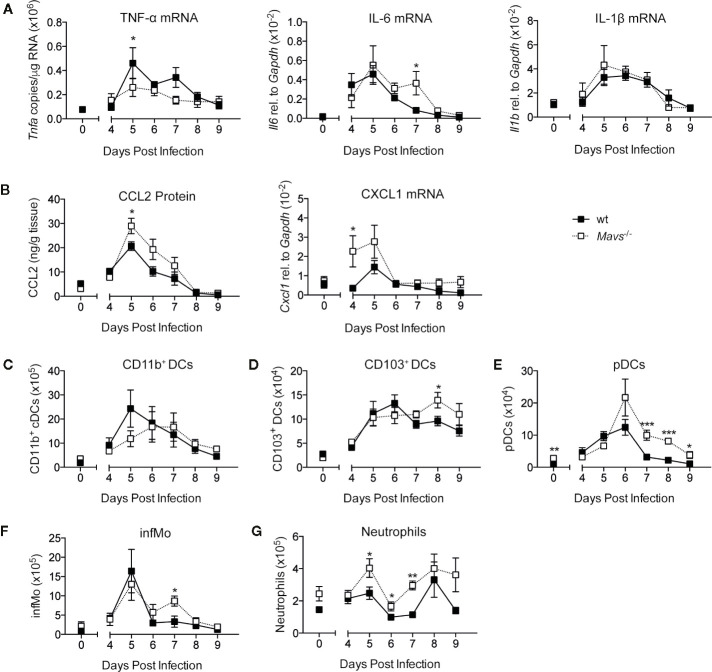
The induction of cytokines, chemokines and cell recruitment on days 4 to 9 of primary RSV infection. **(A)** Gene expression analysis of *Tnfa*, *Il6*, *Il1b*, and **(B)** CCL2 protein levels and *Cxcl1* mRNA were determined at indicated time points in lung tissue from RSV-infected or mock infected (t=0) wt and *Mavs*^−/−^ mice. Total numbers of **(C)** CD11b^+^ DCs, **(D)** CD103^+^ DCs, **(E)** plasmacytoid DCs (pDCs), **(F)** inflammatory monocytes (infMo), and **(G)** neutrophils in the lungs were determined by flow cytometry at indicated time points. Data are represented as mean ± SEM. Data in **(A, B)** are pooled from two independent experiments, n = 5–10. Data in **(C–G)** are representative of at least two experiments with four to five mice per group. Statistical significances of differences between indicated genotypes at each time point was determined by unpaired Student’s *t* test. *P < 0.05; **P < 0.01; ***P < 0.001.

RSV infections are characterized by the rapid (18–24 h following infection) and transient induction of early innate immune mediators such as type I IFNs, IL-6, and TNF-α (3, 5, 6, 17). The production of these cytokines are dependent on signaling *via* MAVS, as previously published (5, 6). To monitor cytokine responses during the later course of primary RSV infection, cytokines and chemokines were measured in lung samples of RSV infected wild type (wt) and *Mavs*^−/−^ mice, starting 4 days post infection (p.i.) up to day 9 p.i. The relative gene expression of *Tnfa*, *Il6*, and *Il1b*, and the protein expression of chemokine CCL2 were all detected on days 5 to 7 p.i. in both wt and *Mavs*^−/−^ mice (**Figures 1A, B**). Notably, this second peak of mediators (days 5–7 p.i.) was smaller than the first peak (18–24 h p.i.) identified in RSV infected wt mice [**Figure 1** and (5, 6)]. The neutrophil chemoattractant CXCL1, was induced more prominently in *Mavs*^−/−^ mice than wt mice during the second peak (days 4–5; **Figure 1B**). This differential expression of the various cytokines and chemokines raised the question about the dynamics of pulmonary innate cells during RSV disease. Therefore, lung cell populations were assessed by flow cytometry from day 4 up to day 9 post RSV infection. Gating was performed as previously described (5). Equivalent to the cytokine and chemokine responses the majority of the identified cell populations (CD103^+^ dendritic cells (DCs), plasmacytoid DCs (pDCs), inflammatory monocytes (CD64^+^ infMo) and neutrophils) showed recruitment into the lung of RSV infected wt and *Mavs*^−/−^ mice from day 4 to day 9 p.i. (**Figures 1C–G**). CD11b^+^ DCs were recruited slightly later in *Mavs*^−/−^ mice and compared to wt mice (**Figure 1C**). CD103^+^ DCs, pDCs and infMo were recruited in both *Mavs*^−/−^ and wt mice but a slightly higher number of these cells was observed in *Mavs*^−/−^ mice at the later time points compared to wt mice (**Figures 1E, F**). Neutrophil numbers in *Mavs*^−/−^ mice were increased over baseline for most time points while wt mice showed neutrophil recruitment on day 8 p.i. (**Figure 1G**). In summary, late responses during RSV infection (days 4–9), including cytokine expression and immune cell recruitment depend on mechanisms other than MAVS signaling and are differently regulated than in the early response (days 0–3) where the cytokine and chemokine responses are mostly dependent on MAVS signaling (5).

### T Cells Are Increased in the Lung of *Mavs*^−/−^ Mice During Primary RSV Infection

**Figure 2:**
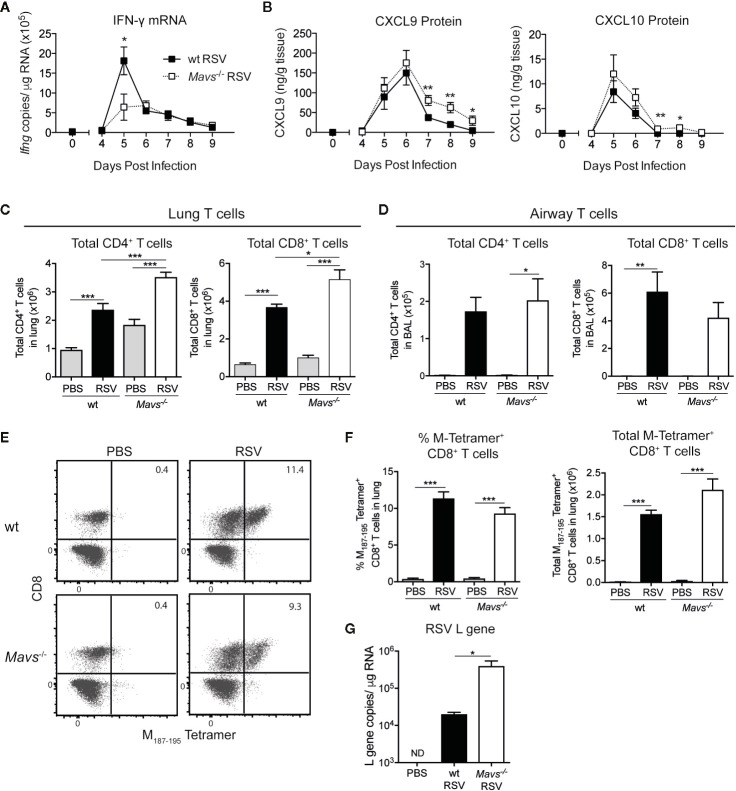
More CD8^+^ and CD4^+^ T cells are detected in lungs of *Mavs*^−/−^ mice compared to wt mice during primary RSV infection. **(A)** Gene expression analysis of *Ifng* was determined by quantitative PCR at indicated time points in lung tissue from RSV-infected or mock-infected (t=0) wt and *Mavs*^−/−^ mice. **(B)** CXCL9 and CXCL10 protein levels were determined by Luminex of lung homogenate at indicated time points. **(C)** Total numbers of CD4^+^ and CD8^+^ T cells in the lung tissue and **(D)** airways of RSV infected or mock-infected (PBS) wt and *Mavs*^−/−^ mice, on day 8 p.i. **(E)** Representative plots of lung tissue M_187-195_ tetramer positive CD8^+^ T cells in wt and *Mavs*^−/−^ mice, on day 8 p.i. Numbers in quadrants represent the percentage of M_187-195_ tetramer positive CD3^+^CD8^+^ T cells in total lung. **(F)** Quantification of frequencies and total M_187-195_ tetramer positive CD8^+^ T cells in lungs, on day 8 p.i. **(G)** Gene expression analysis of RSV L gene in lung tissue was determined by quantitative PCR, on day 8 p.i. Data are represented as mean ± SEM and pooled from two independent experiments, n = 5–10). In **(A, B)** statistical significances of differences between indicated genotypes at each time point was determined by unpaired Student’s *t* test. In **(C, D, F, G)** statistical significances of differences between groups was determined by one-way ANOVA with Tukey’s *post hoc* test. *P < 0.05; **P < 0.01; ***P < 0.001; ND, not detectable.

The induction of appropriate adaptive immune responses and the recruitment of RSV-specific T cells play an essential role in viral clearance, regulating inflammatory responses and disease pathology (2, 13). In previous reports we and others have shown that wt and *Mavs*^−/−^ mice recruit CD4^+^ and CD8^+^ T cells into the lung (5, 6, 11). A quantitative and qualitative analysis of differences in airway and lung T cells during experimental RSV infection and the possible impact of innate immune responses remained elusive. Time course analysis (days 4–9) in the RSV infection model revealed an induction of IFN-γ mRNA from day 5 p.i. in lungs from both wt and *Mavs*^−/−^ mice albeit a more prominent increase was seen in wt mice at day 5 p.i. (**Figure 2A**). T cells are recruited into inflamed tissue by chemokines such as CXCL9 or CXCL10, which are induced by IFNs (18). Both CXCL9 and CXCL10 protein was detected in the lungs of wt and *Mavs*^−/−^ mice and the levels of these chemokines were slightly higher at days 7 to 9 in the *Mavs*^−/−^ mice compared to wt mice (**Figure 2B**). For analysis of T cell responses during RSV infection, wt and *Mavs*^−/−^ mice were sacrificed on day 8 p.i., representing the peak of the T cells response (19, 20). Cells from the lungs and the airways (bronchoalveolar lavage; BAL) were isolated and stained for cell surface markers to phenotypically distinguish different T cell subsets (gating shown in **Supplemental Figure S1A**). Numbers of total CD4^+^ and CD8^+^ T cells were significantly increased in the lung tissue of *Mavs*^−/−^ mice compared to wt mice (**Figure 2C**). However, the recruitment of T cells into the airways showed no differences between *Mavs*^−/−^ and wt mice (**Figure 2D**). RSV-specific CD8^+^ T cells, determined by tetramer staining (gating shown in **Supplemental Figure S1B**) were represented in equal percentages and numbers (**Figures 2E, F**) in the lung tissue of wt and *Mavs*^−/−^ mice, consistent with previous reports from our group and others (5, 6). However, viral clearance was delayed in *Mavs*^−/−^ mice with more RSV L gene copies detected at day 8 p.i. [**Figure 2G** (5, 11)]. This is most likely a result of the absence of a complete innate immune response (5, 6, 11) and an increased viral load might contribute to the heightened recruitment of CD4^+^ and CD8^+^ T cells observed in *Mavs*^−/−^ mice.

### Expression of GzmB in CD8^+^ T Cells Is Enhanced in *Mavs*^−/−^ Mice During Primary Infection With RSV

**Figure 3:**
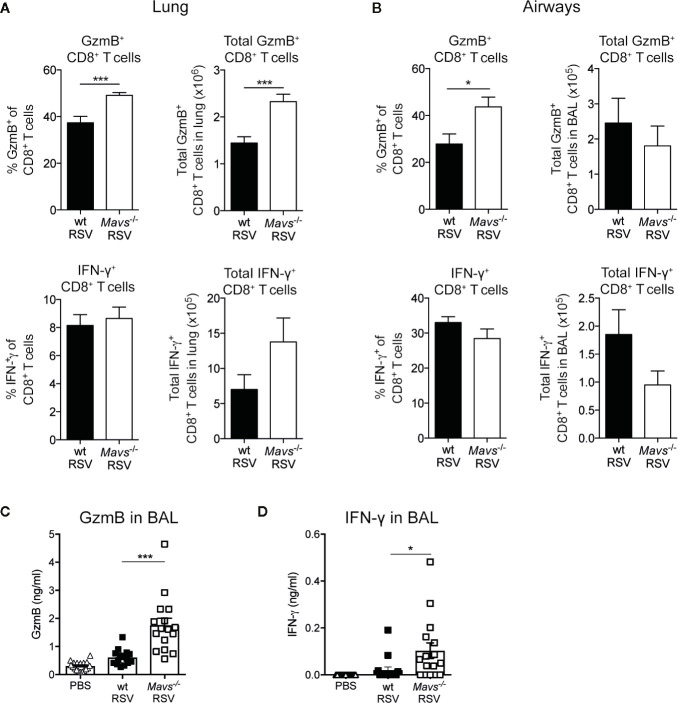
GzmB and IFN-γ producing T cells are increased in *Mavs*^−/−^ mice during primary RSV infection. Percentage and total number of granzyme B (GzmB) and/or IFN-γ producing CD8^+^ T cells in **(A)** lung and **(B)** airways on day 8 in RSV-infected wt and *Mavs*^−/−^ mice, was determined by flow cytometry. **(C)** GzmB and **(D)** IFN-γ levels in BAL fluid of RSV-infected and mock-infected (PBS) mice were determined by ELISA. Data are represented as mean ± SEM. Data in **(A, B)** are pooled from two independent experiments with four to five mice per group (n = 9–10). Statistical significances of differences between indicated genotypes at each time point was determined by unpaired Student’s *t* test. Data in **(C**, **D)** are pooled from three independent experiments with three to five mice per group (n = 14–15). Each symbol represents one individual mouse. Statistical significances of differences between groups were determined by one-way ANOVA with Tukey’s *post hoc* test. *P < 0.05; ***P < 0.001.

During acute viral infection, effector CD8^+^ T cells engage several mechanisms that mediate killing of infected cells and influence antiviral immune responses. For example, the production of cytotoxins, such as granzyme B (GzmB) and perforin, or the expression of surface proteins, like FasL induces programmed-cell death in target cells (14). Additionally, the release of IFN-γ and TNF−α have pleiotropic and immune stimulatory effects (21, 22). We assessed the effector function of lung and airway CD8^+^ T cells after primary encounter with RSV by stimulating them *ex vivo* with RSV M_187-195_ peptide and evaluated their capacity to produce GzmB and IFN-γ, or both, by intracellular staining and flow cytometry analysis (gating shown in **Supplemental Figure S1B**). In the airway and lungs of *Mavs*^−/−^ mice, 40% to 50% of CD8^+^ T cells expressed GzmB, compared to wt mice where 30% to 40% of CD8^+^ T cells stained positive for GzmB (**Figures 3A, B**). Also, total numbers of CD8^+^ GzmB^+^ T cells in the lung tissue was significantly higher in *Mavs*^−/−^ mice at day 8 during primary infection (**Figure 3A**). In terms of the capacity of CD8^+^ T cells to produce IFN-γ, wt and *Mavs*^−/−^ mice displayed no significant differences (**Figures 3A, B**). The same accounted for multifunctional CD8^+^ T cells capable of producing both GzmB and IFN-γ (**Supplemental Figures S2A, B**). This suggested that *Mavs*^−/−^ mice were able to recruit highly activated effector CD8^+^ T cells into the infection site and a large fraction of them were able to secrete effector mediators. Of note, the peptide stimulation did not allow for stimulation of CD4^+^ T cells, which could also be potent producers of IFN-γ. Furthermore, to detect GzmB and IFN-γ; levels *in vivo*, BAL fluids were analyzed. The overall GzmB and IFN-γ; responses were significantly increased in *Mavs*^−/−^ mice at day 8 p.i. compared to wt mice (**Figures 3C, D**). Altogether, our data show that CD4^+^ and CD8^+^ T cell numbers and CD8^+^ T cell effector functions are elevated in the absence of MAVS signaling during primary RSV infection.

### Fewer T Cell in the Lungs After RSV Re-Infection of *Mavs*^−/−^ Mice

During secondary encounter with a pathogen memory T cells within the tissue or recruited from circulation are rapidly activated for defense of the host tissue (15). We hypothesized that the loss of innate immune activation in *Mavs*^−/−^ mice (5, 6, 11) impacts on the quality of the short-term memory responses to RSV. The virus is cleared in both wt and *Mavs*^−/−^ mice after 8 to 9 days post infection (6, 23) so to study short-term memory responses in *Mavs*^−/−^ mice, we re-infected wt and *Mavs*^−/−^ mice 21 days after primary infection with RSV and determined the T cell responses 4 days post re-challenge by flow cytometry (gating strategy see **Supplemental Figure S1A**). To note, *in vivo* labelling of CD45^+^ cells were performed in wt mice at day 4 post re-infection to distinguish T cells residing in the vasculature versus in the lung parenchyma and a majority (70–80%) of the CD4^+^ and CD8^+^ T cells were shown to reside in the lung parenchyma (**Supplemental Figure 3**).

**Figure 4:**
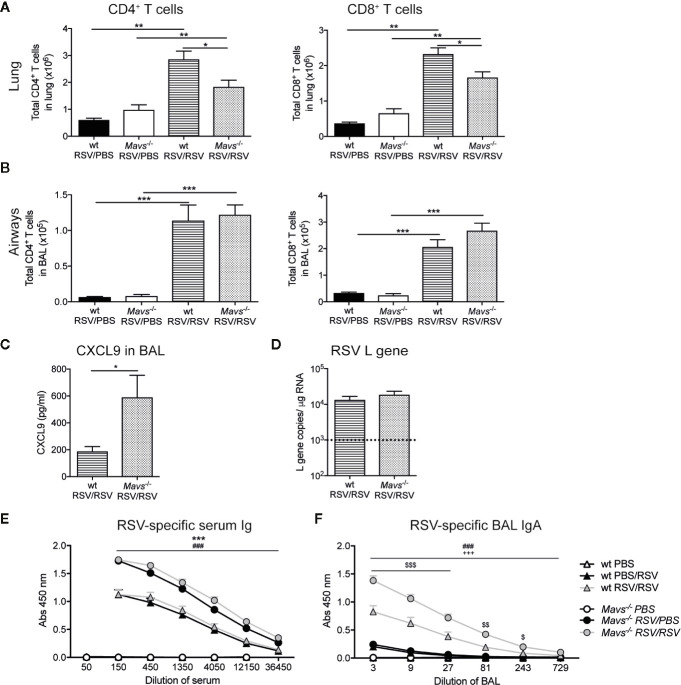
Reduced T cell numbers in the lung of *Mavs*^−/−^ mice after re-infection with RSV. Wt and *Mavs*^−/−^ mice were re-infected with RSV (RSV/RSV) or mock-infected (RSV/PBS) 21 days after primary RSV infection. Four days after secondary infection mice were sacrificed. Total number of CD4^+^ and CD8^+^ T cells in the **(A)** lung and **(B)** airways of wt and *Mavs*^−/−^ mice were determined by flow cytometry. **(C)** Levels of CXCL9 in the BAL fluid of re-challenged mice were determined by Luminex analysis. **(D)** Gene expression analysis of RSV L gene in lung tissue was determined by quantitative PCR, on day 4 post RSV re-infection. Dotted line represents the detection limit. **(E)** RSV-specific serum Ig and **(F)** BAL IgA were determined by ELISA. **(A–C)** Data are represented as mean ± SEM and pooled from two independent experiments, n = 5–10. **(D–F)** Data are represented as mean ± SEM and pooled from three independent experiments with up to five mice per group (PBS n = 7–8 and RSV n = 14–15). Statistical significances of differences between groups were determined by **(A**, **B)** one-way ANOVA with Tukey’s *post hoc* test or **(C, D)** unpaired Student’s *t* test. (**E, F**) Statistical significance of differences between groups at defined dilutions was determined by one-way ANOVA with Turkey’s *post hoc* test. “#” represents statistical significance between wt RSV/RSV and *Mavs*^−/−^ RSV/RSV mice, “*” represents statistical significance between wt RSV/PBS with *Mavs*^−/−^ RSV/PBS mice, “$” represents statistical significance between wt RSV/RSV and wt RSV/PBS, *“*+” represents statistical significance between *Mavs*^−/−^ RSV/RSV and *Mavs*^−/−^ RSV/PBS mice. One symbol p ≤ 0.05; two symbols p ≤ 0.01, three symbols p ≤ 0.001.

Overall, lung CD4^+^ and CD8^+^ T cells were increased in RSV re-challenged mice compared to mice 25 days post RSV primary infection (**Figure 4A**). Between the re-challenged groups, the absolute numbers of CD4^+^ and CD8^+^ T cell in the lung tissue were higher in wt mice compared to *Mavs*^−/−^ mice (**Figure 4A**). Although, the expansion/recruitment of T cells was similar in the airways of both re-infected strains (**Figure 4B**) and the T cell-attracting chemokine CXCL9 was detectable in the BAL fluid of both, wt and *Mavs*^−/−^ mice. Albeit, *Mavs*^−/−^ mice had a two- to three-fold higher level of CXCL9 than wt mice (**Figure 4C**).

To elucidate if this was due to an altered viral load, the number of RSV L gene copies was determined on day 4 after re-infection with RSV. The amount of detectable RSV L gene was low but no differences in lungs from wt and *Mavs*^−/−^ mice were detected (**Figure 4D**). This is probably due to effective neutralization of RSV virions by RSV-specific antibodies (24) as both wt and *Mavs*^−/−^ mice induce neutralizing RSV-specific antibodies. In our study, *Mavs*^−/−^ mice showed higher levels of RSV-specific Ig compared to wt mice (**Figures 4E, F**) while a previous study showed an impaired RSV-specific Ig response in *Mavs*^−/−^ mice (6). Overall, these data suggest a defect in the accumulation of lung memory T cells in the *Mavs*^−/−^ mice.

### Defect in Effector Function of RSV-Specific Memory CD8^+^ T Cells in *Mavs*^−/−^ Mice During Secondary RSV Infection

**Figure 5:**
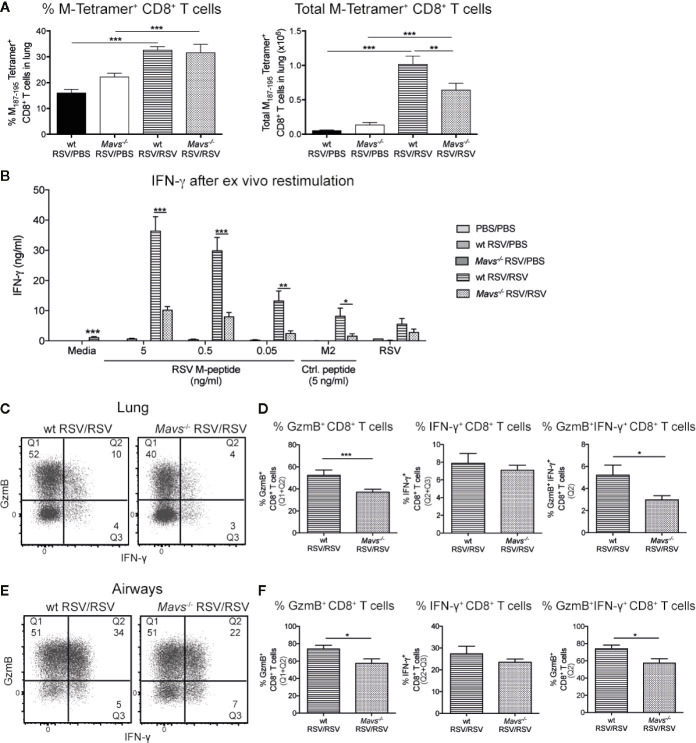
Less potent RSV-specific GzmB and IFN-γ/GzmB producing CD8^+^ T cells in *Mavs*^−/−^ mice during secondary RSV infection. CD8^+^ T cells contribute significantly to IFN-γ; and Granzyme B (GzmB) production during secondary infection with RSV. **(A)** Quantification of frequencies and total M_187-195_ tetramer positive CD8^+^ T cells in lungs, on day 4 post RSV re-infection. **(B)** IFN-γ produced by lung cells after *ex vivo* stimulation with the immunodominant RSV peptide CD8^+^ T cell M_187-195_, the irrelevant peptide M2_82-90_ as control (Ctrl) and RSV (MOI 1). IFN-γ protein levels were determined by ELISA after 72 h. **(C)** Representative flow cytometry plots of CD8^+^ T cells expressing GzmB and/or IFN-γ in the lung tissue of wt and *Mavs*^−/−^ mice re-challenged with RSV (RSV/RSV). The frequencies of GzmB and/or IFN-γ producing cells of the CD8^+^ T cells in the lung are shown in the quadrants and quantified in **(D)**. **(E)** Representative flow cytometry plots of CD8^+^ T cells expressing GzmB and/or IFN-γ in the airways of wt and *Mavs*^−/−^ mice re-challenged with RSV (RSV/RSV). The numbers in the quadrants represent the frequencies of GzmB and/or IFN-γ producing cells of the CD8^+^ T cells in the airways and this is quantified in **(F)**. Data are represented as mean ± SEM and pooled from two independent experiments with four to five mice per group (n = 5–10). In addition, in **(B)** each data point was determined as a mean of duplicate wells. Statistical significances of differences between groups were determined by unpaired Student’s *t* test. In **(B)** only difference between RSV re-infected groups are shown. *P < 0.05; **P < 0.01; ***P < 0.001.

To evaluate the RSV-specific CD8^+^ T cell response three strategies were employed. First, tetramer staining was performed to quantify the frequency and total number of RSV-specific CD8^+^ T cells. *Mavs*^−/−^ mice had fewer lung M-tetramer positive cells compared to wt mice (**Figure 5A**). To confirm these results and quantify total CD8^+^ T cell responses, lung single-cell suspensions were re-stimulated with RSV M_187-195_ peptide at different concentrations (5, 0.5, and 0.05 ng/ml), control peptide RSV M2_82-90_ (5 ng/ml) and RSV (MOI 1) for 3 days and the supernatants assessed for IFN-γ (**Figure 5B**). These data confirmed that *Mavs*^−/−^ mice have fewer RSV-specific CD8^+^ T cells as their lung cells secreted less IFN-γ upon CD8^+^ T cell peptide stimulation than wt lung cells (**Figure 5B**).

We also speculated that, in addition to fewer RSV-specific CD8^+^ T cells, *Mavs*^−/−^ mice additionally exhibit functional defects in the RSV-specific CD8^+^ T cell responses. After a short peptide restimulation, frequency and number of CD8^+^ GzmB^+^ and multifunctional CD8^+^ T cells (IFN-γ and GzmB) in both lung tissue and airways, were significantly reduced in *Mavs*^−/−^ mice compared to wt mice (**Figures 5C–F**). In absolute numbers, a decrease in GzmB^+^ and GzmB^+^IFN-γ;^+^ cells were noted in the lung (**Supplemental Figure 4**). Taken together, our data show that numbers and full functionality of short-term memory CD8^+^ T cells are dependent on signaling *via* MAVS.

## Discussion

RSV infection induces a strong innate immune response *via* the RLR-MAVS pathway with type I IFNs and subsequent IFNAR-signaling identified as central mediators of early pro-inflammatory responses and monocyte recruitment (4–6). However, the effects of the innate immune responses have on the subsequent T cell responses during RSV infection are not fully elucidated. Whether the lack of MAVS signaling influence the immune responses in the later stages of primary RSV infection or if MAVS signaling influence T cell responses during RSV infection was investigated in this study.

Type I IFN secretion comprises the imminent response triggered in infected cells with robust antiviral and immunomodulatory response to contain virus replication and spread (25). During RSV infection, type I IFNs derived from alveolar macrophages (AMs) regulate the early inflammatory and antiviral activity of different lung cell populations (4, 5, 17). This response is completely dependent on MAVS signaling since type I IFNs and many other early mediators are completely absent in *Mavs*^−/−^ mice after RSV infection (5, 6). Also, deficiency in MAVS signaling and/or deficiency in type I IFNs receptor signaling promote susceptibility to RSV-induced disease (4, 5). However, the cytokine and chemokine expression in the lung of wt mice after infection with RSV is characterized by a bi-phasic induction resulting in two peaks of leukocyte infiltration and cytokines and chemokine secretion in the lungs (previously described (26–29) and confirmed in our study). A second peak (days 4–9 p.i.) was detected for the chemokines CCL2, CXCL1, CXCL9 and CXCL10, the pro-inflammatory cytokines TNF-α, IL-6, IL-1β, and IFN-γ; and DCs, CD64^+^ inflammatory monocytes and neutrophils. These observations resembles experimental RSV challenge studies in humans, where a transient induction of the neutrophil chemokine CXCL8 (mouse homologue CXCL1) shortly after inoculation was followed by an additional CXCL8 peak during RSV-induced illness and viral shedding (30). Additionally, IL-8, CCL2, CCL3, and CCL5 were elevated in nasal secretions during symptomatic illness (30). Importantly, this second wave of cytokine and chemokine gene upregulation with cellular infiltration was initiated by mechanism/s independent of the RLR-MAVS pathway. In the later stages of primary RSV infection, apoptotic epithelial cells and immune cells are markedly increased in the lungs (31). It is therefore possible that signaling of endosomal TLR3 and TLR7 in phagocytic cells that clear apoptotic cells containing viral antigen or cells expressing DAMP receptors become increasingly important.

Interestingly, *Mavs*^−/−^ mice showed significant increased influx of CD103^+^ DCs, pDCs, infMo and neutrophils in the respiratory tract [our data and (11)]. Notably, the viral load in the lung of *Mavs*^−/−^ mice was higher than in wt mice (5, 6, 11) and this could drive the increased recruitment of these cell types to the lung. Similarly, influx of monocytes and neutrophils was abundant in autopsy material from infants who died from RSV-induced LRTI (31).

The second peak of cytokines and cellular infiltration in the lung coincides with T cells infiltrating the lungs and also RSV-induced illness characterized by increased airway hyperresponsiveness, airway resistance and weight loss in mice (17, 26). In most infections the activation of the adaptive immune responses and their potent effector functions is required for the final elimination of invasive pathogens. It is commonly believed that the initiation of the adaptive immune system requires innate cytokines, such as type I IFNs (12). Accordingly, it was very surprising to discover similar or slightly increased T cell responses in *Mavs*^−/−^ mice during primary infection [day 8 p.i.; data presented here and (5, 6, 11)]. There are several possible explanations for this. Firstly, the viral load in the lung of *Mavs*^−/−^ mice is higher than in wt mice (5, 6, 11) and this could be driving stronger T cell responses. It is important to note that similar levels of RSV specific CD8^+^ T cells were detected in wt and *Mavs*^−/−^ mice, which could suggest a heightened recruitment of CD8^+^ T cells with other TCR specificities. Second, a MAVS independent mechanism could drive the maturation and antigen presentation capacity of DCs. We have previously shown that mice deficient in MyD88/TRIF and MAVS can mount T cell responses albeit to a reduced level compared to wt mice (32). In addition, Bhoj *et al*. described a migratory DC subset in RSV-infected *Mavs*^−/−^ mice suggested to be involved in cross-presentation of exogenous antigen to CD8^+^ T cells in the draining lymph nodes (6). A specific characterization of this particular DC subset and the specific molecular signals that lead to priming of T cells by DCs remain unclear.

After two to three weeks the majority of activated T cells undergo apoptosis and only a small pool of virus-specific memory T cells are maintained (15, 33). Upon secondary encounter with a pathogen, activation and clonal expansion of the memory T cell pool is crucial for a rapid host defense and survival. We show that activation and/or expansion of short-term memory cells was compromised in the lungs of mice lacking MAVS signaling, which led to a significant impairment in the production of the T cell effector molecules GzmB and IFN-γ. To note, this was observed in the lung but not in the airways. This was not due to a difference in viral load or RSV-specific antibody titers. Interestingly, the *Mavs*^−/−^ mice showed higher RSV-specific Ig responses. The deficiency of short term memory T cells could be the result of a defect in the priming phase of the T cell response that leads to a problem in establishing or retaining a memory T cell pool. Alternatively, MAVS signaling could be necessary during the re-activation of memory T cell in the lungs during RSV secondary infection. To fully re-activate memory T cells, cognate and non-cognate signals (such as type I IFNs, IL-15, or IL-18) are required to rapidly acquire effector functions (34–36). Kohlmeier *et al*. showed that signaling of type I IFNs through the type I IFN receptor (IFNAR) on memory CD8^+^ T cells enhances GzmB production and cytolytic activity in recall responses to influenza virus (34). Therefore, it is possible that the memory T cells formed during primary RSV infection in *Mavs*^−/−^ mice cannot be re-activated due to a lack of essential inflammatory cytokines. Also, the type of memory cells in the lung will be important to evaluate. Resident memory T cells have been shown to be important during RSV infection (37, 38) and the formation and function of these cells in MAVS deficient mice will be important to elucidate in future studies.

In conclusion, our work emphasizes a role for MAVS signaling beyond the initiation of early anti-viral responses that protect from RSV infection. In particular, we identified a critical role for the RLR-MAVS pathway in the numbers and effector functions of the short term memory T cell pool during secondary RSV infection. RSV elicits a short-lived memory response in humans and our data suggest that a weak innate response results in an impaired short-term T cell memory response, especially in the CD8^+^ T cells, which highlights the importance of a balanced immune response to achieve an efficient and long-lasting anti-viral immune response important both during infection and vaccination.

## Data Availability Statement

All datasets presented in this study are included in the article/**Supplementary Material**.

## Ethics Statement

The animal study was reviewed and approved by Imperial College AWERB.

## Author Contributions

MiP designed, performed and analyzed the experiments and wrote the paper. NP, AV, and FK performed specific experiments and reviewed the paper. MaP helped in designing the flow cytometry panel. CJ supervised the project, designed and analyzed the experiments, and wrote the paper. All authors contributed to the article and approved the submitted version.

## Funding

CJ was supported by a Career Development Award from the Medical Research Council (Grant G0800311) and a grant from the Rosetrees Trust and the Stoneygate Trust (M370). MiP was supported by a PhD Fellowship from the National Heart and Lung Institute Foundation (registered charity number 1048073). FK was supported by a Wellcome Trust grant (109058/Z/15/Z) and AV by European Respiratory Society and the Asociación Latinoamericana de Tórax joint long-term Research Fellowship 2019 (Number: LTRF 201901-00546). 

## Conflict of Interest

The authors declare that the research was conducted in the absence of any commercial or financial relationships that could be construed as a potential conflict of interest.
