# Supplementary material for: MAVS Deficiency Is Associated With a Reduced T Cell Response Upon Secondary RSV Infection in Mice
Source: Front Immunol. 2020 Oct 6;11:572747. doi: 10.3389/fimmu.2020.572747 (PMC7573121; doi:10.3389/fimmu.2020.572747)
Supplement: Supplementary file 1 [file DataSheet_1.pdf]

Supplementary Material

a Surface staining

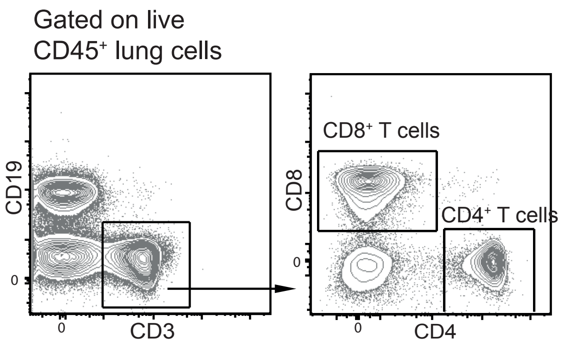

b Intracellular IFN-γ and Granzyme B staining, and M-tetramer staining

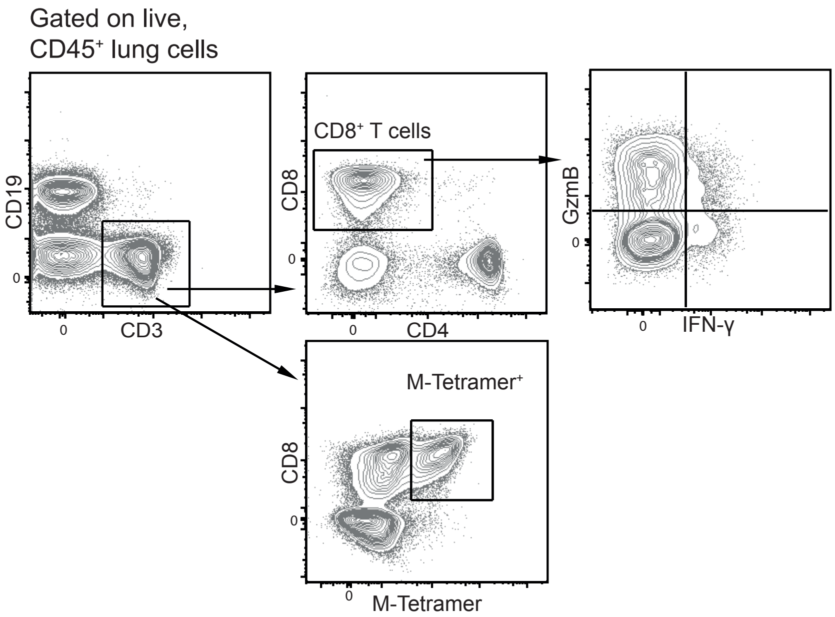

Supplemental Figure 1. Gating strategy to identify lung and BAL T cell populations. Mice were infected with RSV. Cells were obtained from airways by bronchoalveolar lavage and from lung tissue by collagenase digestion and stained for the indicated cell markers. (**a**) After gating on non-debris, singlets, live and $CD45^+$ cells, the gating strategy as shown was used to identify $CD8^+$ and $CD4^+$ T cells. (**b**) After gating on non-debris, singlets, live and $CD45^+$ cells, the gating strategy as shown was used to identify effector molecules produced by $CD8^+$ T cells using intracellular staining for GzmB and IFN-γ. To identify RSV-specific $CD8^+$ T cells, cells were stained with tetramers containing the immunodominant peptide $M_{187\text{-}195}$.

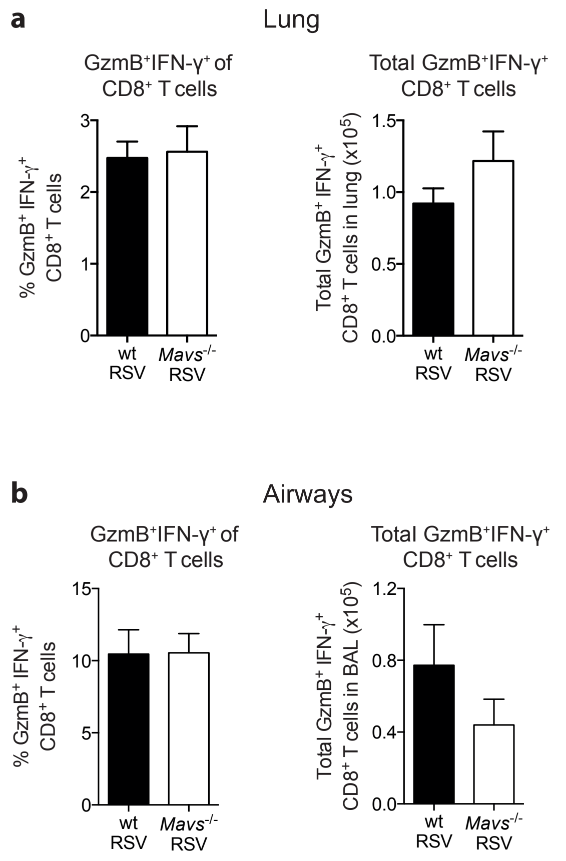

Supplemental Figure 2. Frequency and total number of multifunctional (Granzyme B (GzmB) and IFN-γ producing) $CD8^+$ T cells in (**a**) lung tissue and (**b**) airways on day 8 p.i. in RSV-infected wt and *Mavs*$^{-/-}$ mice, was determined by flow cytometry. Data are represented as mean ± SEM and are pooled from two independent experiments, n=5-10.

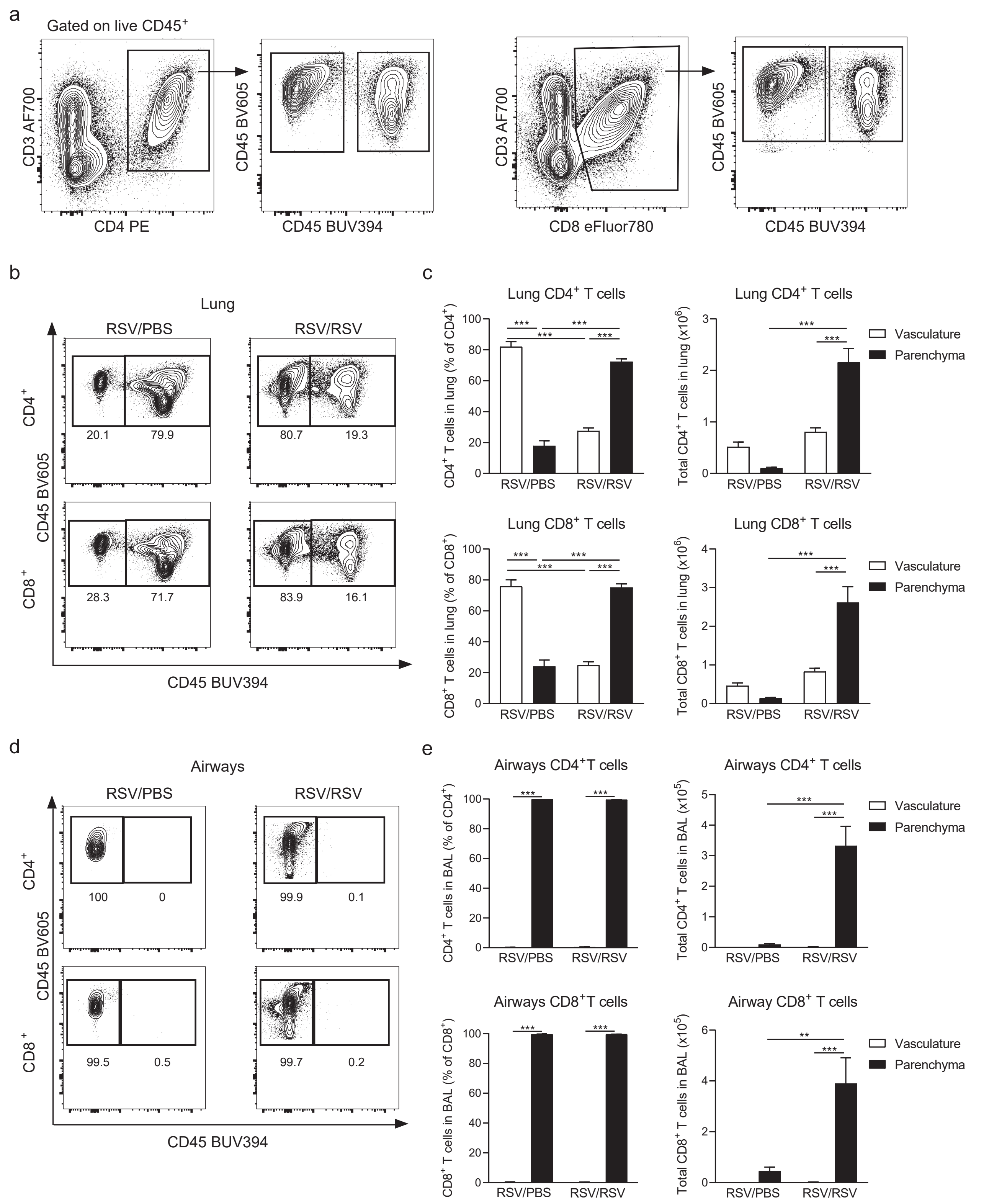

Supplemental Figure 3. C57BL/6 mice were re-infected with RSV (RSV/RSV) or mock-infected (RSV/PBS) 21 days after primary RSV infection. At day 4 post re-infection mice were culled 10 minutes after intravenous injection with anti-CD45 antibody conjugated with BUV395 for discerning intravenous cells ($BUV395^+$ and $BV605^+$) from lung resident cells $BV605^+$. (**a**) Gating strategy for

identification of CD4$^+$ and CD8$^+$ T cells in the parenchyma or vasculature by flow cytometry. (**b**) Representative dot plots for CD4$^+$ and CD8$^+$ T cells harvested from the lung. (**c**) Frequency and total number of CD4$^+$ or CD8$^+$ T cells in the vasculature or lung parenchyma. (**d**) Representative dot plots for CD4$^+$ and CD8$^+$ T cells recovered from BAL. (**e**) Frequency and total number of CD4$^+$ or CD8$^+$ T cells in the airways. Data are presented as mean ± SEM of five mice per group and are representative of two independent experiment. Statistical significances of differences between groups were determined by one-way ANOVA with Tukey's post hoc test. *, P < 0.05; **, P < 0.01; ***, P < 0.001.

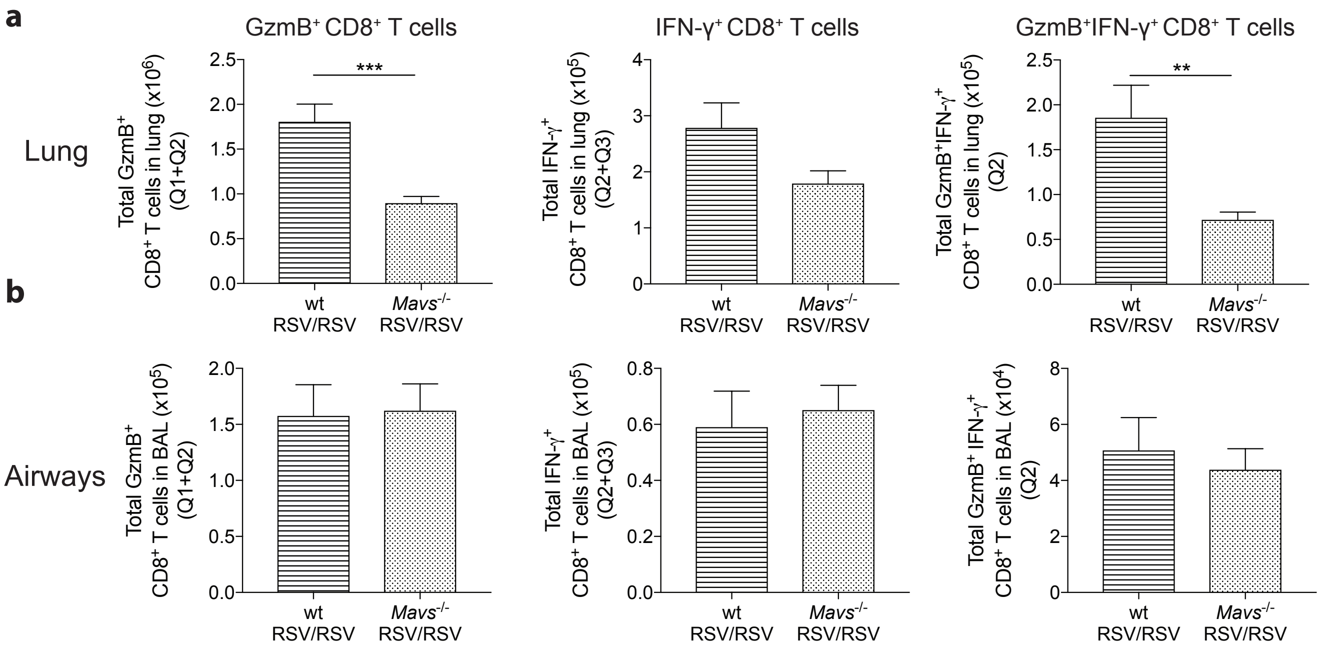

Supplemental Figure 4. Fewer GzmB and IFN-γ/GzmB producing T cells in the lung of *Mavs*$^{-/-}$ mice during secondary RSV infection. Total CD8$^+$ T cells expressing GzmB and/or IFN-γ were quantified in (**a**) the lung tissue or (**b**) airways of wt and *Mavs*$^{-/-}$ mice re-challenged with RSV (RSV/RSV). Data are represented as mean ± SEM and pooled from two independent experiments, n=5-10. Statistical significances of differences between groups were determined by unpaired student's *t* test.
